# Redefining Tumor-Associated Macrophage Subpopulations and Functions in the Tumor Microenvironment

**DOI:** 10.3389/fimmu.2020.01731

**Published:** 2020-08-04

**Authors:** Kaiyue Wu, Kangjia Lin, Xiaoyan Li, Xiangliang Yuan, Peiqing Xu, Peihua Ni, Dakang Xu

**Affiliations:** Faculty of Medical Laboratory Science, Ruijin Hospital, School of Medicine, Shanghai Jiao Tong University, Shanghai, China

**Keywords:** tumor microenvironment, tumor-associated macrophages, multiplexed immunohistochemical staining, single-cell sequencing, spatial transcriptomics

## Abstract

The immunosuppressive status of the tumor microenvironment (TME) remains poorly defined due to a lack of understanding regarding the function of tumor-associated macrophages (TAMs), which are abundant in the TME. TAMs are crucial drivers of tumor progression, metastasis, and resistance to therapy. Intra- and inter-tumoral spatial heterogeneities are potential keys to understanding the relationships between subpopulations of TAMs and their functions. Antitumor M1-like and pro-tumor M2-like TAMs coexist within tumors, and the opposing effects of these M1/M2 subpopulations on tumors directly impact current strategies to improve antitumor immune responses. Recent studies have found significant differences among monocytes or macrophages from distinct tumors, and other investigations have explored the existence of diverse TAM subsets at the molecular level. In this review, we discuss emerging evidence highlighting the redefinition of TAM subpopulations and functions in the TME and the possibility of separating macrophage subsets with distinct functions into antitumor M1-like and pro-tumor M2-like TAMs during the development of tumors. Such redefinition may relate to the differential cellular origin and monocyte and macrophage plasticity or heterogeneity of TAMs, which all potentially impact macrophage biomarkers and our understanding of how the phenotypes of TAMs are dictated by their ontogeny, activation status, and localization. Therefore, the detailed landscape of TAMs must be deciphered with the integration of new technologies, such as multiplexed immunohistochemistry (mIHC), mass cytometry by time-of-flight (CyTOF), single-cell RNA-seq (scRNA-seq), spatial transcriptomics, and systems biology approaches, for analyses of the TME.

## Introduction

The tumor microenvironment (TME), which refers to the structure of tumor tissue containing stromal cells (including immune cells, connective tissue cells, and vascular components), is crucial in tumor progression and metastasis. A close association between inflammation and the TME has been established in recent years, although the link was first noted in the nineteenth century (1). Currently, inflammation in the TME is generally considered a hallmark of cancer (2), reflecting that inflammatory cells interact with tumor cells to influence the progression of tumors. Among the diverse inflammatory cells infiltrating the TME, macrophages, which are termed tumor-associated macrophages (TAMs), including both resident macrophages and circulating monocytes recruited to the TME, are predominant elements (3).

The role of TAMs in the TME, which is critical to current TAM-targeted strategies, remains to be uncovered due to the intricate heterogeneity of macrophages. Preclinical and clinical data show a close relationship between high infiltration of TAMs and a poor prognosis in most types of tumors (4), such as pancreatic ductal adenocarcinoma (PDAC) (5), glioblastoma (6), and bladder cancer (7). On the other hand, TAM infiltration has also been found to be associated with a favorable prognosis in some cases, such as in ovarian cancer (8) and colorectal cancer (9). Such different outcomes can be attributed to not only the distinct cancer types but also some intra-tumoral factors, such as the TAM distribution in the TME. For example, some studies on non-small cell lung cancer (NSLSC) reported that increased infiltration of TAMs in tumor islets was associated with a good prognosis, whereas increased levels of TAMs in the tumor stroma were found to be associated with a poor prognosis (10, 11). These findings may indicate the inter- and intra-tumoral heterogeneity of TAMs, which may relate to the ontogeny, activation status, and localization of TAMs in the TME. To discriminate the distinct roles of TAMs among various conditions, TAM subsets and their functions in the TME urgently need to be redefined.

In this review, we will summarize the current understanding of the dual roles that TAMs play in the TME and highlight the inter- and intra-tumoral heterogeneity of TAMs, thus emphasizing the necessity of further investigating and redefining TAM subpopulations with distinct functions. The integration of some novel and powerful technologies as a work flow to analyze the heterogeneity of TAMs will also be discussed, including multiplexed immunohistochemistry (mIHC), mass cytometry by time-of-flight (CyTOF), single-cell RNA-seq (scRNA-seq), and spatial transcriptomics.

## Characteristics of TAMs

**Figure 1:**
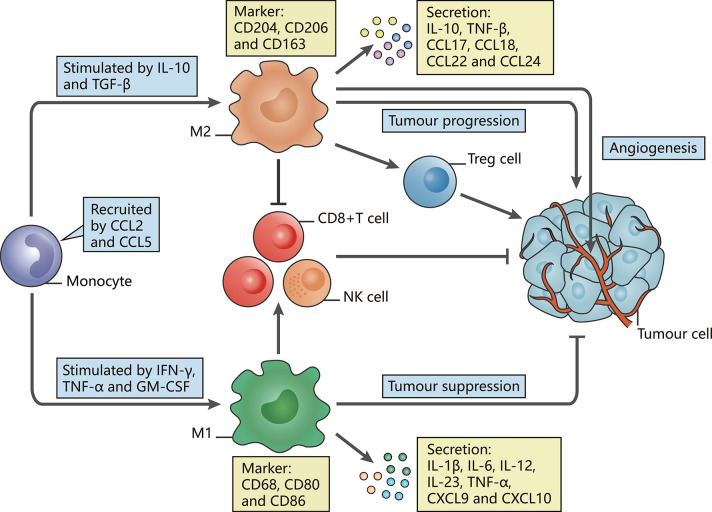
The polarization of TAMs and their characteristics. The figure displays a general principle of polarized M1-like and M2-like phenotypes. M1-like and M2-like phenotypes represent two extremes of TAM polarization and display distinct functions. In response to different stimuli in the TME, TAMs undergo M1-like, or M2-like activation. M1-like TAMs are stimulated by IFN-γ, TGF-α, or GM-CSF, express CD68, CD80, and CD86, secrete IL-1β, IL-6, IL-12, IL-23, CXCL9, and CXCL10, and exert anti-tumor effects. In contrast, M2-like TAMs are activated by IL-10 or TGF-β, express CD163, CD204, and CD206, secrete IL-10, TNF, CCL17, CCL18, CCL22, and CCL24 and promote tumor progression.

Signals in the TME may impact the diversity and function of TAMs, leading to dual roles for TAMs in tumor progression that can be summarized as tumor-promoting and tumor-suppressing activities (12). In accordance with the commonly accepted theory proposed by Mills' team, TAMs can be mainly classified into the antitumor M1 phenotype (classically activated state) and the pro-tumor M2 phenotype (alternatively activated state), reflecting the Th1-Th2 polarization of T cells (13, 14). Once TAMs derived from peripheral blood monocytes are recruited to the TME by tumor-secreted attractants, they undergo M1-like or M2-like activation in response to various stimuli (Figure 1).

Induced by interferon-γ (IFN-γ) (15), tumor necrosis factor α (TNF-α) and granulocyte-macrophage colony-stimulating factor (GM-CSF) (16, 17), M1-like TAMs are involved in activating Th1-type immune responses as they have a high capacity for antigen presentation. They produce nitric oxide (NO), reactive oxygen species (ROS) and pro-inflammatory cytokines such as interleukin (IL)-1β, IL-6, IL-12, IL-23, C-X-C motif chemokine (CXCL) 9, CXCL10, TNF-α, and major histocompatibility complex (MHC) molecules (18–24). The expression of surface proteins, including CD68, CD80, and CD86 (25), and the intracellular protein suppressor cytokine signaling 3 (SOCS3) can also be upregulated (26). Through secretion of the described factors, M1-like TAMs function as the main forces in innate host defense and kill tumor cells, thus suppressing tumors.

In contrast, M2-like TAMs, which are generated under the influence of several cytokines such as IL-10 and transforming growth factor (TGF)-β, activate Th2-type immune responses and promote tumorigenesis and development (27). They may mainly promote upregulation of the expression of anti-inflammatory cytokines and chemokines, including IL-10, TGF-β, CC chemokine ligand (CCL) 17, CCL18, CCL22, and CCL24 (24). Such secretion is involved in tumor invasion and metastasis. Surface proteins, such as CD206 (mannose receptor-1), CD204 and CD163 (macrophage scavenger receptors), are also overexpressed (28). These M2-like TAMs have critical roles in facilitating epithelial-mesenchymal transition (EMT), angiogenesis and immunosuppression (29, 30). Moreover, M2-like TAMs are one of the factors hampering the efficacy of chemotherapy and radiotherapy through suppression of CD8^+^ T cell function, leading to tumor progression and poor outcomes (28, 31, 32). Additionally, we summarize several current markers linked with clinical outcomes that appear in studies of TAMs in different tumor types (Table 1).

**Table 1:** TAMs markers correlated with clinical outcomes and functions.

**TAM marker**	**Tumor type**	**Level**	**Overall function**	**Clinical outcome**	**Function**	**References**
CD68	Breast	High	Pro-tumor	Reduced OS Increased tumor stage and size	Promote invasion and lymphatic metastasis of breast cancer	(33)
CD68	Gastric	High	Pro-tumor	Reduced OS Lymph node metastasis Higher TNM stage	Enhance tumor growth and aggressiveness	(34)
CD68	Colorectal	High	Anti-tumor	Improved OS Reduced tumor budding	Counter the aggressive tumor budding phenotype	(35)
CD68	Prostate	High	Anti-tumor	Improved DFS Lower TNM stage	Express NOS2 and TNF-α Contribute to tumor cell cytotoxicity	(36)
CD163	Breast	High	Pro-tumor	Reduced RFS and DSS	Promote cancer cells migration and intravasation into both blood and lymphatic vessels	(37)
CD163	HNSCC	High	Pro-tumor	Poor OS and PFS	Promote tumor progression	(38)
CD163	Pancreatic	High	Pro-tumor	Reduced OS	Upregulate CD59 expression on cancer cells Protect cancer cells from complement-dependent cytotoxicity	(39)
CD163	Colorectal	High	Anti-tumor	Lower tumor grade Reduced lymph node metastasis	Counter cancer cell invasion	(35)
CD204	Breast	High	Pro-tumor	Poor OS, RFS and DMFS	Promote tumor cell proliferation, migration and invasion	(40)
CD204	LADC	High	Pro-tumor	Reduced DFS Advanced tumor stage Lymphovascular invasion Lymph node metastasis	Associated with tumor aggressiveness	(41)
CD204	Oesophageal	High	Pro-tumor	Reduced OS	Elevate the PD-L1 expression in cancer cells Promote tumor cell invasion and migration	(42)
CD206	Ovarian	High	Pro-tumor	Lymphatic invasion	Upregulate expressions of MMP-2, MMP-9 and MMP-10 Enhance ovarian cancer cells invasion via TLRs signaling pathway	(43)
CD206	OSCC	High	Pro-tumor	Reduced DSS and PFS Higher clinical stage Cervical nodal metastasis	Promote proliferation and invasion in OSCC via EGF production	(44)
Folate receptor β	Pancreatic	High	Pro-tumor	Reduced OS	Promote angiogenesis, hematogenous metastasis Upregulate expression of VEGF	(45)
Wnt5a+CD68+/CD68+	Colorectal	Ratio high	Pro-tumor	Reduced RFS and OS Higher TNM stage	Secrete IL-10 to induce M2 polarization Promote tumor proliferation, migration and invasion (Wnt5a+CD68+ macrophages)	(46)
Galectin-9 and CD68	Bladder	High coexpression	Pro-tumor	Poor OS and RFS	Correlated with increasing numbers of Tregs and decreasing numbers of CD8+T cell Related to reduced cytotoxic molecules, enhanced immune checkpoints or immunosuppressive cytokines.	(47)
CD163+CD204+	OSCC	High	Pro-tumor	Reduced PFS	Promote T-cell apoptosis and immunosuppression via IL-10 and PD-L1	(48)
CD68++CD163+	Gastric	High^*^	Anti-tumor	Increased OS and RFS	Clear dead cells and remodel tissue	(49)
CD68 and HLA-DR	NSCLC	High coexpression	Anti-tumor	Increased survival time	Prevent progression of NSCLC	(50)
CD68 and HLA-DR	NSCLC	High coexpression	Anti-tumor	Increased DSS	Exhibit antitumoral functions	(51)
CD68 and NOS2	Gastric	High coexpression	Anti-tumor	Preferent survival	Immuno-stimulatory	(52)
CD86	ICC	High	Anti-tumor	Longer median overall OS	Promote tumor cytotoxicity Amplify Th1 responses	(53)
NOS2	Colorectal	High	Anti-tumor	Increased RFS Improved survival in a stage dependent manner	Provide a positive feedback loop in anti-tumor response Tumor prevention	(54)

*Wnt5a, Wnt family member 5A; NOS2, nitric oxide synthase-2; HNSCC, head and neck squamous cell carcinoma; LADC, lung adenocarcinoma; OSCC, oral squamous cell carcinoma; NSCLC, Non-small cell lung cancer; ICC, intrahepatic cholangiocarcinoma; OS, overall survival; TNM, tumor-node-metastasis; DFS, disease-free survival; RFS, relapse free survival; DS S, disease-specific survival; PFS, progression-free survival; DJ'v:IFS, distant metastasis survival*.

* *Only in the effective density (effective density: the number of TAM that had a tumor cell within a 10 f!m radius)*.

However, the characteristics of TAMs summarized in Figure 1 mostly correspond to conditions *in vitro*, and TAMs are not precisely divided into the M1 and M2 phenotypes *in vivo*, reflecting the insufficiency of this principle in understanding the comprehensive functions of TAMs due to the heterogeneity defined to date. Markers of the M1 and M2 phenotypes can be co-expressed on an individual cell (55). These markers also show defects when applied to differentiate the antitumor M1-like and pro-tumor M2-like phenotypes. For example, CD163 and CD206 are common M2-associated markers, but TAMs highly expressing CD163 and CD206 in gastrointestinal tumors or ovarian ascites were found to be functionally equivalent to M1-like TAMs with regard to stimulating T cell activity (56). The explanation for the link between TAM definition and function relies heavily on the M1-M2 paradigm, which may have greatly distorted our perception. Using current widely accepted biomarkers, whether TAMs actually exert pro-tumor or antitumor functions is unclear (57). Our understanding of functional markers may be far too simple to decipher the complex activation of TAMs in the TME.

## Exploring the Marked Diversity of TAMs

### Ontogeny of Monocytes and TAMs

Historically, macrophages in the TME were thought to originate from circulating monocyte precursors in the bone marrow (BM), responding to various tissue damage signals. In the search for TAM progenitors in mouse mammary tumors, studies show that tumor-infiltrating monocytes are almost exclusively distinguished by high expression of Ly6C, which serves as a principal marker of mouse monocytes. These Ly6C^high^ monocytes contribute to TAMs continuously and renew all non-proliferating TAM populations, constituting a heterogeneous myeloid fraction including M1-like MHC-II^high^ and M2-like MHC-II^low^ TAM subpopulations (58–60). Recent studies argued that recruited macrophages originated from both the BM and the spleen and suggested a minor splenic contribution to the main proportion of TAMs derived from the BM by utilizing a lineage-tracing analysis of fluorescent spleen- and BM-derived monocytes (61, 62). However, several studies have revealed that a group of macrophages reside in tissues beginning in the early embryonic phase (63, 64), further validating the coexistence of macrophages with different origins (65).

The origin and maintenance of these tissue-resident macrophages (TRMs) is controversial. TRMs were initially thought to originate from circulating monocytes. Recently, adult TRMs have been shown to derive from the continuous wave of embryonic and adult haematopoiesis, and the contribution to each TRM population is tissue specific (66, 67). Using parabiosis and genetic fate-mapping methods, studies have reported that TRMs in some tissues, such as the brain, are maintained locally and continue to undergo self-renewal throughout adult life with minimal contributions from circulating monocytes, while research on BM-derived mononuclear cells has indicated that TRMs in other tissues may have a relatively high monocyte contribution characterized by distinct increases at a tissue-specific speed under steady- and inflammatory-state conditions (66, 68–70). These observations suggest that the origin of TRMs is controlled under both inflammatory and stable conditions, exhibiting tissue-specific and inflammation-specific characteristics. To further identify the monocytic source of TRMs in various conditions, a study used a fate-mapping model with Ms4a3 as the specific gene expressed by granulocyte/monocyte progenitor cells to effectively track monocytes and granulocytes but not lymphocytes or tissue dendritic cells. As a result, the contribution of monocytes to the TRM pool was quantified, showing variations during homeostasis and inflammation in different models (71).

The relative contributions of monocyte-derived TAMs and TRMs in tumor models have also been revealed. Recent cell tracking studies have illustrated at the molecular level that recruited macrophages predominate in the TME because a significant decrease in TAM abundance is often followed by blockade of CC chemokine receptor 2 (the receptor for CCL2, which plays a pivotal role in the recruitment of TAMs) in most cases (72). In research on glioblastoma, inflammatory monocytes/macrophages have been revealed to express both CCR2 and CX3CR1, but microglia express only CX3CR1. Due to such distinct molecular identification, multiparameter flow cytometry analyses have further validated that the relative proportion of more than 85% of the TAMs within tumors are BM-derived macrophages, whereas resident microglia account for the remaining approximate proportion of 15% (73). For the purpose of future functional studies, more evidence for definitive identification of monocyte-derived TAMs and TRMs is required.

Moreover, attention has been gradually drawn to the questionable dictation of functions driven by multiple origins of TAMs. The interplay between TAMs with different origins remains to be elucidated, but studies in mouse models can provide evidence to some extent. Notably, several studies have discussed TRMs and monocyte-derived TAMs in terms of distinct overall functions and genomic differences. TRMs in a mouse model of PDAC were shown to promote PDAC progression with fibrosis-modulating functions, while impairment of circulating monocytes alone had limited impacts (74). Monocyte-derived TAMs have also been suggested to play a stronger role in antigen presentation, whereas TRMs exhibit a profibrotic transcriptional signature, indicating their role in the production and remodeling of molecules in the extracellular matrix (74). Such distinct functions were confirmed in another case of lung cancer, where TRMs were significantly correlated with tumor cell growth *in vivo*, while accumulation of monocyte-derived TAMs led to enhanced tumor dissemination (75). To evaluate differences at the genomic level, a study on glioma using transgenic mice models validated that glioma TAMs expressed Arg1 (an M2 marker) shortly after trafficking into tumors, the expression of which was stimulated relatively later by microglia (76), which may reveal different responses of TAMs with distinct origins to tumor growth. Collectively, these findings may jointly suggest a potential influence of TAM origin on functional changes, which requires further exploration.

However, the differences between mouse and human monocytes, such as the repertoire of surface receptors, must be emphasized (14). The classification of mouse monocyte subsets relies on the differential expression of Ly6C, while human monocytes can mainly be characterized by the expression of CD14 and CD16 into distinct subpopulations of CD14^+^CD16^−^and CD14^lo^CD16^+^ monocytes (60, 77, 78). CD14^+^CD16^−^ monocytes have been proposed to share similarities with Ly6C^hi^ mouse monocytes in the expression patterns of certain molecules. Meanwhile, CD14^lo^CD16^+^ human monocytes are counterparts to Ly6C^lo^ mouse monocytes (79). A study compared the gene expression profiles of monocyte subsets and found some conversely expressed molecules between matched subsets of the two species, such as CD36, CD9, and TREM-1. Peroxisome proliferator activated receptor (PPAR) has also been validated to be prominently expressed in mouse monocytes, which is absent in humans (80). Additionally, specific cytokines regulating cells vary in mice and humans, which also contribute to the differences between the two species (81). Considering the functional differences in monocyte subsets in phagocytic capacity, which is regarded as one of the cardinal features of blood monocytes, patterns of receptors involved in the uptake of apoptotic cells and other phagocytic cargo were also shown to differ in monocyte subsets of humans and mice (80). Most of our current knowledge depends on mouse models, and whether the origins of human TRMs match those of mice remains to be considered. Comparisons of distinct types of monocyte-derived macrophages with TRMs in humans revealed a lack of specific markers indicative of the subset of origin, resulting in incomplete knowledge of their functions among various cancer types (24, 82, 83), which may indicate the need for further investigation to address the possible different monocyte lineages in humans.

### Plasticity of TAMs

TAMs can further display remarkable plasticity within the TME and switch from one phenotype to another (84). However, the M1/M2 paradigm represents two extreme activation states of TAMs, which may neglect that the adaption driven by environmental signals in the TME is flexible rather than static. These environmental cues are mainly stimulated by tumor cells, immune cells, and the extracellular matrix (85). TAMs show the ability to reversibly respond to specific stimuli in the TME, transforming antitumor M1-like and pro-tumor M2-like phenotypes during the immune response under certain conditions. Such plasticity also results in diverse subpopulations of TAMs.

The pro-inflammatory M1-like phenotype in the TME may evolve into the M2-like phenotype following tumor progression, thus exerting a tumor-supporting influence (86). Chemokines such as CXCL12 can be highly secreted from monocytes during tumor progression and then facilitate the transition from M1-like to M2-like TAMs, forming a proangiogenic and immunosuppressive response with upregulation of M2 inducers (87). Recently, the adaptative ability of TAMs has been further validated in pre-clinical models and clinical trials, and the regulation of TAM polarization to enhance antitumor functions has been successfully stimulated. The PI3K-γ pathway and colony-stimulating factor 1 (CSF-1)/colony-stimulating factor 1 receptor (CSF-1R) expression are generally considered important in the polarization of M2-like TAMs (88, 89). A study reported that dual blockade of the PI3K-γ pathway and CSF-1/CSF-1R resulted in a switch from an M2-like state to an M1-like state in PDAC models (90), leading to a reduction in immunosuppressive macrophage numbers and stimulation of a CD8^+^ T cell response. Inhibition of CSF-1/CSF-1R alone has achieved similar effects in other models of glioblastoma (91), melanoma (92), and rhabdomyosarcoma (93). A CD40 agonist has also been reported to stimulate the transformation of a pro-tumor M2-like state into an antitumor M1-like state in a PDAC mouse model, enhancing antitumor immune responses (94). Signal transducer and activator of transcription (STAT) 3/STAT6 can reportedly direct tumor-promoting macrophage polarization. A small-molecule inhibitor of STAT3 significantly reduced M2-like polarization in a case of malignant glioma, while TAMs in STAT6-deficient mice displayed an M1-like phenotype, enhancing antitumor immunity (95). Additional targets, such as CCL5-CCR5, IL-12, histone deacetylases (HDACs), and tyrosine-protein kinase receptor 2 (TIE2), have been explored to reprogramme TAMs to suppress tumor growth (96). Taken together, these results suggest that suppressing the tumor-promoting functions of TAMs can elevate antitumor activities and reverse the immunosuppressive status in the TME.

The plasticity of macrophages highlights macrophage reprogramming as an attractive therapeutic strategy to inhibit tumor progression, enabling these cells to adapt their function to meet the needs of antitumor defense. To better understand the activated status of TAMs within the TME, further studies on specific markers to differentiate the distinct functions of antitumor and pro-tumor TAMs are in high demand.

### Intra-Tumoral Heterogeneities of TAMs

In addition to inter-tumoral heterogeneity, several factors may contribute to the intra-tumoral heterogeneity of TAMs, especially tumor hypoxia and the distribution of TAMs in the TME.

Hypoxia, which often develops within the TME due to an imbalance between oxygen supply and demand caused by abnormal vasculature, acts as a powerful attractant of TAMs (97). TAMs can be continuously elicited to hypoxic regions through elevated expression of hypoxia-inducible factor (HIF)-1α, a key transcription factor that regulates hypoxia-induced gene expression. HIF-1α upregulates CXC receptor 4 (CXCR4) in monocytes/macrophages and the specific ligand CXCL12 and also induces the chemotactic responsiveness among these reactants (98, 99). Moreover, TAM migration may be inhibited under the influence of hypoxia, resulting in TAM accumulation (98). Consequently, TAMs are recruited and maintained in hypoxic compartments with increased expression of chemoattractants, thus fostering tumor progression (100). Strong tumor hypoxia and high-density hypoxic TAMs have been associated with poor survival, highlighting the clinical significance of hypoxia (101).

Notably, hypoxia contributes significantly to the pro-tumoral functions of MHC-II^lo^ M2-like TAMs by altering gene expression profiles rather than directly influencing TAM differentiation (102). TAMs are prone to develop a pro-angiogenic phenotype under the influence of hypoxia, which is involved in metabolism, angiogenesis, and metastasis. When assessing intra-tumoral localization depending on the level of hypoxia, a study selectively labeled MHC-II^lo^ M2-like TAMs and MHC-II^hi^ M1-like TAMs and found that MHC-II^lo^ TAMs predominated in hypoxic regions, while MHC-II^hi^ TAMs resided in less hypoxic areas. These hypoxia-oriented TAMs achieve a proangiogenic response not only by directly upregulating angiogenic molecules, especially VEGF-A, which is a potent pro-angiogenic factor (60), but also through upregulation of angiogenic modulators such as matrix metalloproteinase (MMP) 7 (103). Additionally, hypoxia-oriented TAMs may also suppress T cell activation through upregulation of IL-10 and negative checkpoint regulators such as PD-L1 (104). A recent study also showed an increased level of indoleamine 2, 3-dioxygenase (IDO) when co-culturing macrophages with hepatoma cells under hypoxic conditions, limiting the proliferation of cytotoxic T cells as well as expanding Treg cells (105). In contrast, impeding TAM migration to hypoxic areas may result in a more antitumoral macrophage phenotype and reduced tumor growth. A study established that the Sema3A/Neuropilin-1 signaling axis controlled the entry of TAMs into hypoxic regions, and that specifically blunting this pathway enhanced antitumor immunity and alleviated angiogenesis, thus inhibiting tumor growth and metastasis (104). This phenomenon demonstrates the interaction between TAMs and tumor hypoxia, highlighting the partial intra-tumoral heterogeneity determined by hypoxia.

Similar to hypoxia, different histological distributions of TAMs have also been correlated with distinct tumor progression according to a large number of experimental studies in mice, which are usually divided into the tumor nest (TN), tumor stroma (TS) and invasive tumor margin (TM). In a study evaluating the distribution of TAMs and the associated survival rate in gastric cancer (GC), increased CD163^+^ TAM accumulation in the TS and TM was found to be closely related to tumor progression, whereas the relationship between CD163^+^ TAMs in the TN and tumor progression was not as close as that between CD163^+^ TAMs and the TS or TM (106). The prominent role that TAMs in the TS play in tumor progression over those in the TN has also been validated in other types of tumors (107, 108). Moreover, specific localization of TAMs may also impact how they affect tumor growth; for instance, TN-associated macrophages are more pro-angiogenic than macrophages in the TS in breast cancer (109). These functional variations of TAMs may be attributed to their histological locations within the TME and the intra-tumoral heterogeneity of TAMs.

Overall, the functions of TAM subsets exhibit significant intra-heterogeneity in the TME. TAM subsets with distinct functions in the TME must be redefined. An improved understanding of how TAM subsets are influenced by intraregional conditions such as hypoxia and histological distributions will certainly benefit related therapeutic approaches.

## Emerging Technologies for Analyzing Functional Biomarkers in Subsets of TAMs

To further analyze functional biomarkers of TAMs in the TME, high-resolution information is needed to investigate distinct TAM subtypes, which can be obtained by utilizing some distinctive, newly emerging technologies, including mIHC, CyTOF, high-throughput scRNA-seq, and spatial transcriptomics (28).

### mIHC

Along with downstream quantitative image analysis, mIHC represents a powerful tool to visualize and analyze complex cell-to-cell and cell-to-stroma interactions within tumors (110). An increasing number of automated digital pathology systems are being designed for mIHC analysis, such as HALO from Indica Labs (Corrales, NM), which enables tissue segmentation with artificial intelligence and quantifies various histopathological changes as an outstanding image analysis platform (111). Updated information regarding recent technological advances can be found in some reviews (112).

### Mass Cytometry

CyTOF is a next-generation platform for single-cell assessment that overcomes the spectral limitation by replacing fluorophores with metal isotope labels for probes (such as antibodies and RNA probes) (113). Despite its dependence on preselected markers and loss of spatial information, CyTOF contributes substantially to a more comprehensive understanding of cellular phenotypic signatures with the quantification of multiple surface and intracellular proteins, which has been widely applied to explore the phenotypic complexity of microenvironments, such as those of lung adenocarcinoma (114) and diffuse astrocytomas (115). Useful protocols for CyTOF analysis can be referenced in several detailed reviews (116, 117).

### Bulk RNA-seq and scRNA-seq

Compared with bulk RNA-seq, scRNA-seq focuses more on cell heterogeneity, which may address TAM complexity via an unbiased analysis of cells based on transcriptomic profiles and greatly revolutionizes transcriptomic studies. To date, numerous scRNA-seq technologies have been developed for single-cell transcriptomic studies, which are specifically designed for full-length transcripts and the 3′-end or 5′-end of the transcripts. Advanced scRNA-seq methods can provide unprecedented opportunities to comprehensively explore the expression dynamics of both protein-coding and non-coding RNAs at the single-cell level (118). In addition to sequencing, subsequent computational data analyses are critical because scRNA-seq is associated with higher dropouts and nosier data than bulk RNA-seq due to a lower amount of initial material (119). Some reviews have summarized distinct software and applications available for various research purposes (120, 121).

### Spatial Transcriptomics

Recently developed spatial transcriptomics can be complementary to provide detailed visualized spatial information (122). This method can not only offer high-resolution *in situ* gene expression profiles and reveal the molecular genealogy of tissue lineages but also define continuous temporal and spatial pluripotency states, thus identifying the networks of molecular determinants driving lineage specification and tissue organization. Relevant protocols are available in some references (123), and several applications are specialized for spatial transcriptomics, such as ST Spot Detector (124) and ST viewer (125).

## Integrated Strategies to Redefine the Subpopulations and Functions of TAMs

**Figure 2:**
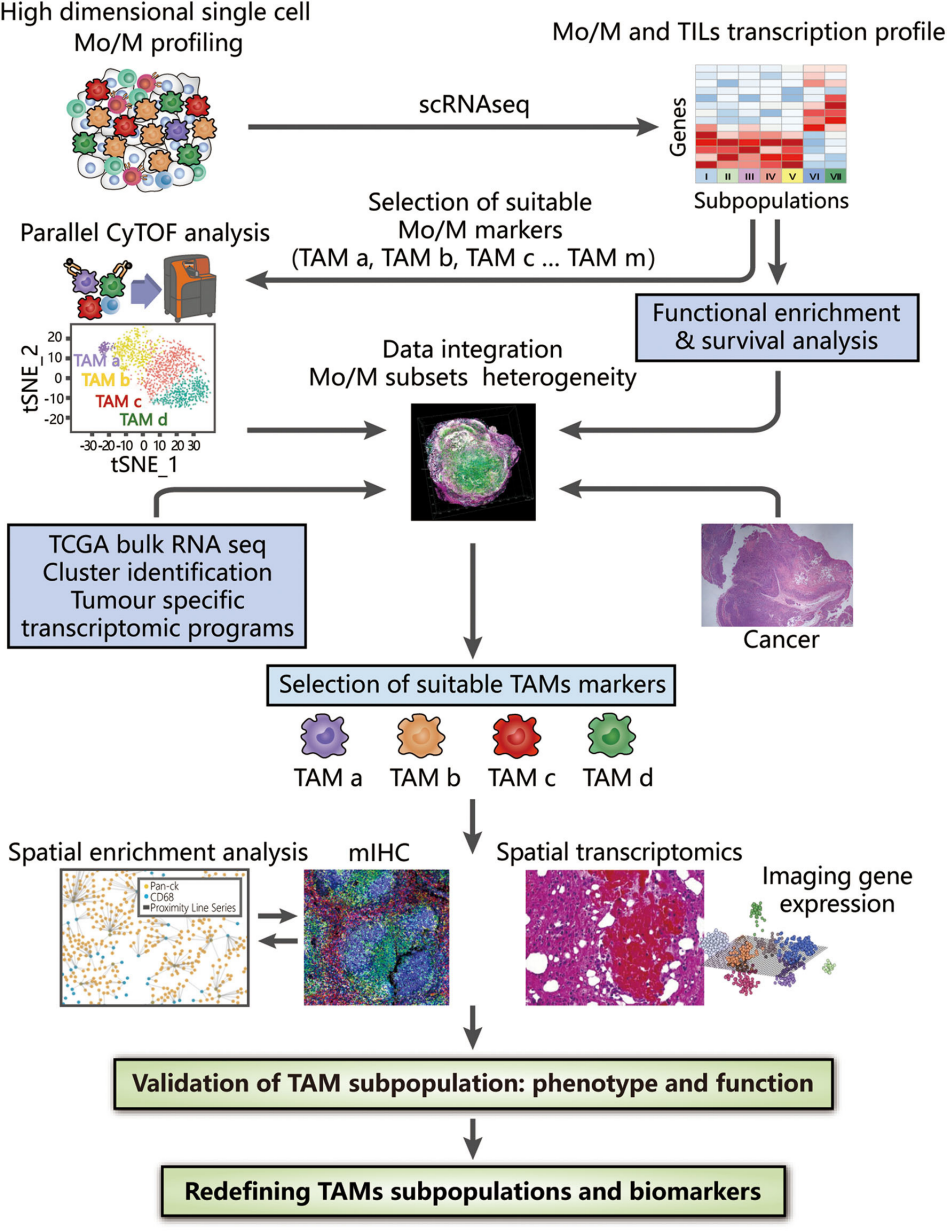
Integrated strategies to redefine the classification of TAMs. High-dimensional analysis of TAMs supported by CyTOF and scRNA-seq, along with bioinformatic approaches (including dimension reduction tools and cluster analysis), provides an overview of surface protein and gene expression, thus contributing to the identification of TAM subsets at the proteomic and transcriptomic levels. Clusters of interest can then be selected depending on either different compositions or distinct functions among identified TAM subpopulations, which are associated with their histopathological characteristics in tissue samples and clinical significance confirmed by survival analysis. By combining bulk RNA-seq data obtained from TCGA and tumor-specific transcriptomic programme, the heterogeneity of TAMs can be further analyzed to provide evidence for the selection of suitable TAM markers. Based on these markers, the spatial distribution in the TME obtained by mIHC and spatial transcriptomics facilitate subsequent generation of the complete landscape in tumor tissues and deconvolution of cell-state relationships, benefiting a deeper understanding of the associations between the functions and phenotypes of TAMs. The integrated use of these technologies strongly reveals the inter- and intra-tumoral heterogeneity of TAMs, potentially redefining TAMs with valuable biomarkers.

To overcome the limitations of applying a single method, the integration of novel methods may provide a preferable solution that may be complementary when characterizing TAMs. The use of multiple techniques has already been applied to study cell heterogeneity and highlight the utility of integrated strategies, such as assessing all haematopoietic cells by scRNA-seq and CyTOF (126), revealing the landscape of immune cells in hepatocellular carcinoma with two different kinds of scRNA-seq (127), and studying the infiltration of cells in PDAC with scRNA-seq and spatial transcriptomics (128). We will further describe a more detailed methodology in proteomic, transcriptomic, spatial, and functional dimensions to examine TAM diversity at the single-cell level, thus generating a complete landscape of TAMs in the TME (Figure 2).

### Identifying TAM Subtypes at the Proteomic and Transcriptomic Levels With CyTOF and scRNA-seq

To identify TAM subtypes at either the proteomic or transcriptomic level, using CyTOF and scRNA-seq together can reveal the diversity of TAMs in detail (126). The gene expression profile generated by scRNA-seq can guide the selection of specific markers of monocytes/macrophages for CyTOF analyses to explore TAM subpopulations at the proteomic level following similar data processing procedures. This combined application also serves as an alternative to further investigate whether any correlation exists between protein and gene expression. The complementary integration of CyTOF and scRNA-seq may potentially show an overlap, thus generating meticulous and complete profiles of both the phenotypes and transcriptomes of TAMs.

### Analyzing Different Compositions or Overall Functional Differences in Identified TAM Subpopulations Among Varying Histopathological Conditions

To distinguish TAM subpopulations with distinct functions, analyses can be conducted based on either different compositions or overall functions through functional enrichment analysis, differential gene expression analysis and survival analysis (126, 129).

Functional enrichment analysis annotates possible immunosuppressive or antitumor functions and pathways of the proteins or genes in highlighted clusters (130). An analysis can be easily performed through the DAVID website, which is one of the most frequently employed enrichment analysis approaches (131). With identified survival-related subpopulations of TAMs through survival analysis, we can then highlight the markers of these clusters and conduct more specific functional studies on them.

Examining the possible overall difference in functions among diverse conditions is usually most feasible and may lead to the discovery of clusters enriched in specific pathways associated with functions of either tumor promotion or suppression. Moreover, we may then explore and discuss the differentially expressed biomarkers among these identified clusters, thus contributing to research on potential biological differences.

### Associating With Bulk RNA-seq Profiles From TCGA and Tumor-Specific Transcriptomic Programme

Notably, scRNA-seq serves as a powerful tool to reveal TAM heterogeneity but may be limited by insufficient specimens. Bulk RNA-seq profiles provided by The Cancer Genome Atlas (TCGA), which contains large cohorts of samples and reports the averaged gene expression across a broad range of cells in various tumor types, can supplement previous results obtained by scRNA-seq, enabling further identification of TAM subpopulations and validation of bulk transcriptomic data. Scrutinizing previous findings with a wider range of statistics in TCGA is essential to reach more reliable conclusions regarding functional associations (132).

Moreover, a tumor-specific transcriptomic programme can be applied to identify relationships between somatic mutation alterations, which are considered a main cause of cancer and differentially expressed genes within tumors. Such a programme can also focus on networks of tumor-specific genes to measure the activation status of corresponding pathways, further indicating the inter- and intra-tumoral heterogeneity together with previous approaches.

### Mapping Distinct TAM Subpopulations and Deconvolving Cell-State Relationships Within the TME

Supplementary application of mIHC or spatial transcriptomics can be employed to further determine how the local microenvironment may impact cell functions and the cell state. A computer-supported workflow can be generally followed to quantitatively characterize the spatial heterogeneity of TAMs in the TME with different metrics, including cell density, cell/cluster, the mean distance, and the cluster area. In this workflow, image analysis algorithms are first employed to identify and locate TAMs in the patient tissue sample, mapping out the coordinates of each cell of interest and facilitating successive analysis of spatial point patterns and morphology. On the one hand, intra-tumoral heterogeneity can be determined directly through application of the spatial point process model in the full-point mode sliding window. On the other hand, morphological analysis extracts information to illustrate inter-cellular interactions and related geometric properties of the cell clusters. Moreover, calculated indicators of each cluster further guide the establishment and validation of systems biology models for immune-oncology research as well as their associated prognostic outcomes.

Meanwhile, spatial transcriptomic technology is a favorable alternative to spatial techniques designed to evaluate the gene expression profile of single cells, which complements missing spatial information in scRNA-seq. Using histological tissue sections and spatial barcoding to analyze gene expression, as well as downstream analysis, we can generate sample clusters that correspond to well-defined morphological features and unbiasedly detect the spatial distributions of marker genes in tumor tissue samples.

Overall, the integrated application of high-throughput techniques overcomes the limitations of each method and results in a complementary profile on the basis of phenotype, transcriptome, and infiltration status (133). Although TAMs have been proposed as novel therapeutic targets and several treatments to eradicate or modulate TAMs are being evaluated (134), a major gap exists in our current understanding of diverse TAM subsets, biomarkers, and their functions. This methodological strategy focuses on examining specific TAM heterogeneity in primary tumors as well as their metastases in multiple dimensions and benefits researchers studying how ontogeny, activation status and localization dictate macrophage biomarkers, undoubtedly showing a promising ability to discriminate TAM subsets with specific biomarkers and to establish firm correlations between particular TAM populations and clinical outcomes. Such research into heterogeneous TAM biology will be highly relevant to the design of new and specific antitumor therapies targeting TAMs to achieve therapeutic effects as well as possible.

## Summary and Outlook

TAMs, the key components in the TME, are related to tumor invasion and metastasis and have shown emerging potential as new targets for cancer immunotherapy (135). As thoroughly discussed, the ontogeny, plasticity, and inter- and intra-tumoral heterogeneity of TAMs have complicated defining the exact function of TAMs from the currently limited understanding of TAM phenotypes, emphasizing the necessity of redefining the subtypes and functions of TAMs in the TME.

In this review, we provide a framework to decipher differences among TAM functional phenotypes and their composition in the TME, establishing an all-encompassing analysis that includes the phenotypes, transcriptomes, spatial distributions, and functions of TAM subsets with the integration of novel techniques to offer more detailed and complementary information. This approach may show unique advantages that bypass the limitations of each technique in studies.

Through our strategy, we (1) identify TAM subpopulations at the phenotypic and transcriptomic levels in a single-cell solution; (2) associate TAM subpopulations with histopathological and clinical characteristics, identifying TAM subtype-specific markers for spatial studies; (3) generate a complete landscape of the TME and map TAM subpopulations to deconvolve cell-state relationships; and (4) highlight valuable TAM subpopulations. This review offers important insight into redefinition of TAMs with functional biomarkers. From a therapeutic standpoint, our strategy offers the possibility of precisely targeting TAM subpopulations with distinct antitumor functions. However, more joint efforts are warranted to generate a common vision of TAM heterogeneity.

## Author Contributions

DX designed the structure of this article. KW, KL, and DX wrote the manuscript. XY reviewed the manuscript. XL, PX, and PN made substantial and intellectual contributions to the work. All authors approved the article for publication.

## Conflict of Interest

The authors declare that the research was conducted in the absence of any commercial or financial relationships that could be construed as a potential conflict of interest.

## References

[1] Balkwill F Mantovani A. Inflammation and cancer: back to virchow? Lancet. (2001) 357:539–45. 10.1016/S0140-6736(00)04046-0 11229684

[2] Mantovani A Sica A. Macrophages, innate immunity and cancer: balance, tolerance, and diversity. Curr Opin Immunol. (2010) 22:231–7. 10.1016/j.coi.2010.01.009 20144856

[3] Murray PJ Allen JE Biswas SK Fisher EA Gilroy DW Goerdt S. Macrophage activation and polarization: nomenclature and experimental guidelines. Immunity. (2014) 41:14–20. 10.1016/j.immuni.2014.06.008 25035950 PMC4123412

[4] Zhang QW Liu L Gong CY Shi HS Zeng YH Wang XZ. Prognostic significance of tumor-associated macrophages in solid tumor: a meta-analysis of the literature. PLoS ONE. (2012) 7:e50946. 10.1371/journal.pone.0050946 23284651 PMC3532403

[5] Huang X He C Lin G Lu L Xing K Hua X. Induced CD10 expression during monocyte-to-macrophage differentiation identifies a unique subset of macrophages in pancreatic ductal adenocarcinoma. Biochem Biophys Res Commun. (2020) 524:1064–71. 10.1016/j.bbrc.2020.02.042 32070494

[6] Zhang X Chen L Dang WQ Cao MF Xiao JF Lv SQ. CCL8 secreted by tumor-associated macrophages promotes invasion and stemness of glioblastoma cells via ERK1/2 signaling. Lab Invest. (2019) 100:619–29. 10.1038/s41374-019-0345-3 31748682

[7] Wu H Zhang XX Han DL Cao JL Tian JQ. Tumour-associated macrophages mediate the invasion and metastasis of bladder cancer cells through CXCL8. Peer J. (2020) 8:e8721. 10.7717/peerj.8721 32201645 PMC7073239

[8] El-Arabey AA Denizli M Kanlikilicer P Bayraktar R Ivan C Rashed M. GATA3 as a master regulator for interactions of tumor-associated macrophages with high-grade serous ovarian carcinoma. Cell Signal. (2020) 68:109539. 10.1016/j.cellsig.2020.109539 31935430

[9] Edin S Wikberg ML Oldenborg PA Palmqvist R. Macrophages good guys in colorectal cancer. Oncoimmunology. (2013) 2:e23038. 10.4161/onci.23038 23524684 PMC3601174

[10] Feng PH Yu CT Wu CY Lee MJ Lee WH Wang LS. Tumor-associated macrophages in stage IIIA pN2 non-small cell lung cancer after neoadjuvant chemotherapy and surgery. Am J Transl Res. (2014) 6:593–603. 10.1158/1538-7445.AM2014-2556 25360223 PMC4212933

[11] Dai F Liu L Che G Yu N Pu Q Zhang S. The number and microlocalization of tumor-associated immune cells are associated with patient's survival time in non-small cell lung cancer. BMC Cancer. (2010) 10:220. 10.1186/1471-2407-10-220 20487543 PMC2880994

[12] Salmaninejad A Valilou SF Soltani A Ahmadi S Abarghan YJ Rosengren RJ. Tumor-associated macrophages: role in cancer development and therapeutic implications. Cell Oncol. (2019) 42:591–608. 10.1007/s13402-019-00453-z 31144271 PMC12994359

[13] Mills CD Kincaid K Alt JM Heilman MJ Hill AM. M-1/M-2 macrophages and the Th1/Th2 paradigm. J Immunol. (2000) 164:6166–73. 10.4049/jimmunol.164.12.6166 10843666

[14] Biswas SK Mantovani A. Macrophage plasticity and interaction with lymphocyte subsets: cancer as a paradigm. Nat Immunol. (2010) 11:889–96. 10.1038/ni.1937 20856220

[15] Billiau A Matthys P. Interferon-gamma: a historical perspective. Cytokine Growth Factor Rev. (2009) 20:97–113. 10.1016/j.cytogfr.2009.02.004 19268625

[16] Fleetwood AJ Lawrence T Hamilton JA Cook AD. Granulocyte-macrophage colony-stimulating factor (CSF) and macrophage CSF-dependent macrophage phenotypes display differences in cytokine profiles and transcription factor activities: implications for CSF blockade in inflammation. J Immunol. (2007) 178:5245–52. 10.4049/jimmunol.178.8.5245 17404308

[17] Zhu J Zhi Q Zhou BP Tao M Liu J Li W. The role of tumor associated macrophages in the tumor microenvironment: mechanism and functions. Anticancer Agents Med Chem. (2016) 16:1133–41. 10.2174/1871520616666160520112622 27198986

[18] Fujiwara N Kobayashi K. Macrophages in inflammation. Curr Drug Targets Inflamm Allergy. (2005) 4:281–6. 10.2174/1568010054022024 16101534

[19] Porta C Riboldi E Ippolito A Sica A. Molecular and epigenetic basis of macrophage polarized activation. Semin Immunol. (2015) 27:237–48. 10.1016/j.smim.2015.10.003 26561250

[20] Schultze JL Schmidt SV. Molecular features of macrophage activation. Semin Immunol. (2015) 27:416–23. 10.1016/j.smim.2016.03.009 27049460

[21] Lewis CE Pollard JW. Distinct role of macrophages in different tumor microenvironments. Cancer Res. (2006) 66:605–12. 10.1158/0008-5472.CAN-05-4005 16423985

[22] Zheng X Turkowski K Mora J Brne B Seeger W Weigert A. Redirecting tumor-associated macrophages to become tumoricidal effectors as a novel strategy for cancer therapy. Oncotarget. (2017) 8:48436–52. 10.18632/oncotarget.17061 28467800 PMC5564660

[23] Jeannin P Paolini L Adam C Delneste Y. The roles of CSFs on the functional polarization of tumor-associated macrophages. Febs J. (2018) 285:680–99. 10.1111/febs.14343 29171156

[24] Biswas SK Allavena P Mantovani A. Tumor-associated macrophages: functional diversity, clinical significance, and open questions. Semin Immunopathol. (2013) 35:585–600. 10.1007/s00281-013-0367-7 23657835

[25] de Sousa JR de Sousa RP Aarao TL Dias LB Jr. Carneiro FR Fuzii HT. *In situ* expression of M2 macrophage subpopulation in leprosy skin lesions. Acta Trop. (2016) 157:108–14. 10.1016/j.actatropica.2016.01.008 26827741

[26] Huang X Li Y Fu M Xin HB Polarizing macrophages *in vitro*. In: Rousselet G, editor. Macrophages: Methods and Protocols. New York, NY: Springer New York (2018). p. 119–26. 10.1007/978-1-4939-7837-3_12 PMC8875934 29761394

[27] Griess B Mir S Datta K Teoh-Fitzgerald M. Scavenging reactive oxygen species selectively inhibits M2 macrophage polarization and their pro-tumorigenic function in part, via Stat3 suppression. Free Radic Biol Med. (2020) 147:48–60. 10.1016/j.freeradbiomed.2019.12.018 31863907 PMC10035558

[28] Jayasingam SD Citartan M Thang TH Zin AAM Ang KC Ch'ng ES. Evaluating the polarization of tumor-associated macrophages into M1 and M2 phenotypes in human cancer tissue: technicalities and challenges in routine clinical practice. Front Oncol. (2020) 9:1512. 10.3389/fonc.2019.01512 32039007 PMC6992653

[29] Zhang Q Huang F Yao Y Wang J Wei J Wu Q. Interaction of transforming growth factor-β-Smads/microRNA-362–3 p/CD82 mediated by M2 macrophages promotes the process of epithelial-mesenchymal transition in hepatocellular carcinoma cells. Cancer Sci. (2019) 110:2507–19. 10.1111/cas.14101 31215741 PMC6676115

[30] DeNardo DG Ruffell B. Macrophages as regulators of tumour immunity and immunotherapy. Nat Rev Immunol. (2019) 19:369–82. 10.1038/s41577-019-0127-6 30718830 PMC7339861

[31] Cassetta L Pollard JW. Targeting macrophages: therapeutic approaches in cancer. Nat Rev Drug Discov. (2018) 17:887–904. 10.1038/nrd.2018.169 30361552

[32] Pathria P Louis TL Varner JA. Targeting tumor-associated macrophages in cancer. Trends Immunol. (2019) 40:310–27. 10.1016/j.it.2019.02.003 30890304

[33] Yang J Li X Liu X Liu Y. The role of tumor-associated macrophages in breast carcinoma invasion and metastasis. Int J Clin Exp Pathol. (2015) 8:6656–64. 26261547 PMC4525881

[34] Su CY Fu XL Duan W Yu PW Zhao YL. High density of CD68+ tumor-associated macrophages predicts a poor prognosis in gastric cancer mediated by IL-6 expression. Oncol Lett. (2018) 15:6217–24. 10.3892/ol.2018.8119 29616104 PMC5876426

[35] Koelzer VH Canonica K Dawson H Sokol L Karamitopoulou-Diamantis E Lugli A. Phenotyping of tumor-associated macrophages in colorectal cancer: impact on single cell invasion (tumor budding) and clinicopathological outcome. Oncoimmunology. (2016) 5:e1106677. 10.1080/2162402X.2015.1106677 27141391 PMC4839334

[36] Shimura S Yang G Ebara S Wheeler TM Frolov A Thompson TC. Reduced infiltration of tumor-associated macrophages in human prostate cancer: association with cancer progression. Cancer Res. (2000) 60:5857–61. 11059783

[37] Klingen TA Chen Y Aas H Wik E Akslen LA. Tumor-associated macrophages are strongly related to vascular invasion, non-luminal subtypes, and interval breast cancer. Hum Pathol. (2017) 69:72–80. 10.1016/j.humpath.2017.09.001 28923419

[38] Troiano G Caponio VCA Adipietro I Tepedino M Santoro R Laino L. Prognostic significance of CD68(+) and CD163(+) tumor associated macrophages in head and neck squamous cell carcinoma: a systematic review and meta-analysis. Oral Oncol. (2019) 93:66–75. 10.1016/j.oraloncology.2019.04.019 31109698

[39] Zhang R Liu Q Peng J Wang M Gao X Liao Q. Pancreatic cancer-educated macrophages protect cancer cells from complement-dependent cytotoxicity by up-regulation of CD59. Cell Death Dis. (2019) 10:836. 10.1038/s41419-019-2065-4 31685825 PMC6828776

[40] He Y Zhou S Deng F Zhao S Chen W Wang D. Clinical and transcriptional signatures of human CD204 reveal an applicable marker for the protumor phenotype of tumor-associated macrophages in breast cancer. Aging. (2019) 11:10883–901. 10.18632/aging.102490 31799941 PMC6932883

[41] Sun Y Xu S. Tumor-associated CD204-positive macrophage is a prognostic marker in clinical stage I lung adenocarcinoma. Biomed Res Int. (2018) 2018:8459193. 10.1155/2018/8459193 29850577 PMC5926519

[42] Yagi T Baba Y Okadome K Kiyozumi Y Hiyoshi Y Ishimoto T. Tumour-associated macrophages are associated with poor prognosis and programmed death ligand 1 expression in oesophageal cancer. Eur J Cancer. (2019) 111:38–49. 10.1016/j.ejca.2019.01.018 30822683

[43] Ke X Zhang S Wu M Lou J Zhang J Xu T. Tumor-associated macrophages promote invasion via Toll-like receptors signaling in patients with ovarian cancer. Int Immunopharmacol. (2016) 40:184–95. 10.1016/j.intimp.2016.08.029 27608303

[44] Haque A Moriyama M Kubota K Ishiguro N Sakamoto M Chinju A. CD206(+) tumor-associated macrophages promote proliferation and invasion in oral squamous cell carcinoma via EGF production. Sci Rep. (2019) 9:14611. 10.1038/s41598-019-51149-1 31601953 PMC6787225

[45] Kurahara H Takao S Kuwahata T Nagai T Ding Q Maeda K. Clinical significance of folate receptor beta-expressing tumor-associated macrophages in pancreatic cancer. Ann Surg Oncol. (2012) 19:2264–71. 10.1245/s10434-012-2263-0 22350599

[46] Liu Q Yang C Wang S Shi D Wei C Song J. Wnt5a-induced M2 polarization of tumor-associated macrophages via IL-10 promotes colorectal cancer progression. Cell Commun Signal. (2020) 18:51. 10.1186/s12964-020-00557-2 32228612 PMC7106599

[47] Qi Y Chang Y Wang Z Chen L Kong Y Zhang P. Tumor-associated macrophages expressing galectin-9 identify immunoevasive subtype muscle-invasive bladder cancer with poor prognosis but favorable adjuvant chemotherapeutic response. Cancer Immunol Immunother. (2019) 68:2067–80. 10.1007/s00262-019-02429-2 31720813 PMC11028176

[48] Kubota K Moriyama M Furukawa S Rafiul H Maruse Y Jinno T. CD163(+)CD204(+) tumor-associated macrophages contribute to T cell regulation via interleukin-10 and PD-L1 production in oral squamous cell carcinoma. Sci Rep. (2017) 7:1755. 10.1038/s41598-017-01661-z 28496107 PMC5431876

[49] Huang YK Wang M Sun Y Di Costanzo N Mitchell C Achuthan A. Macrophage spatial heterogeneity in gastric cancer defined by multiplex immunohistochemistry. Nat Commun. (2019) 10:3928. 10.1038/s41467-019-11788-4 31477692 PMC6718690

[50] Ma J Liu L Che G Yu N Dai F You Z. The M1 form of tumor-associated macrophages in non-small cell lung cancer is positively associated with survival time. BMC Cancer. (2010) 10:112. 10.1186/1471-2407-10-112 20338029 PMC2851690

[51] Rakaee M Busund LR Jamaly S Paulsen EE Richardsen E Andersen S. Prognostic value of macrophage phenotypes in resectable non-small cell lung cancer assessed by multiplex immunohistochemistry. Neoplasia. (2019) 21:282–93. 10.1016/j.neo.2019.01.005 30743162 PMC6369140

[52] Pantano F Berti P Guida FM Perrone G Vincenzi B Amato MM. The role of macrophages polarization in predicting prognosis of radically resected gastric cancer patients. J Cell Mol Med. (2013) 17:1415–21. 10.1111/jcmm.12109 24283947 PMC4117554

[53] Sun D Luo T Dong P Zhang N Chen J Zhang S. CD86(+)/CD206(+) tumor-associated macrophages predict prognosis of patients with intrahepatic cholangiocarcinoma. Peer J. (2020) 8:e8458. 10.7717/peerj.8458 32002338 PMC6982414

[54] Edin S Wikberg ML Dahlin AM Rutegard J Oberg A Oldenborg PA. The distribution of macrophages with a M1 or M2 phenotype in relation to prognosis and the molecular characteristics of colorectal cancer. PLoS ONE. (2012) 7:e47045. 10.1371/journal.pone.0047045 23077543 PMC3471949

[55] Chong BF Tseng LC Hosler GA Teske NM Zhang S Karp DR. A subset of CD163+ macrophages displays mixed polarizations in discoid lupus skin. Arthritis Res Ther. (2015) 17:324. 10.1186/s13075-015-0839-3 26568320 PMC4644297

[56] Elliott LA Doherty GA Sheahan K Ryan EJ. Human tumor-infiltrating myeloid cells: phenotypic and functional diversity. Front Immunol. (2017) 8:86. 10.3389/fimmu.2017.00086 28220123 PMC5292650

[57] Yang M McKay D Pollard JW Lewis CE. Diverse functions of macrophages in different tumor microenvironments. Cancer Res. (2018) 78:5492–503. 10.1158/0008-5472.CAN-18-1367 30206177 PMC6171744

[58] Auffray C Sieweke MH Geissmann F. Blood monocytes: development, heterogeneity, and relationship with dendritic cells. Ann Rev Immunol. (2009) 27:669–92. 10.1146/annurev.immunol.021908.132557 19132917

[59] Van Overmeire E Stijlemans B Heymann F Keirsse J Morias Y Elkrim Y. M-CSF and GM-CSF receptor signaling differentially regulate monocyte maturation and macrophage polarization in the tumor microenvironment. Cancer Res. (2016) 76:35–42. 10.1158/0008-5472.CAN-15-0869 26573801

[60] Movahedi K Laoui D Gysemans C Baeten M Stange G Van den Bossche J. Different tumor microenvironments contain functionally distinct subsets of macrophages derived from Ly6C(high) monocytes. Cancer Res. (2010) 70:5728–39. 10.1158/0008-5472.CAN-09-4672 20570887

[61] Shand FHW Ueha S Otsuji M Koid SS Shichino S Tsukui T. Tracking of intertissue migration reveals the origins of tumor-infiltrating monocytes. Proc Natl Acad Sci USA. (2014) 111:7771–6. 10.1073/pnas.1402914111 24825888 PMC4040600

[62] Cortez-Retamozo V Etzrodt M Newton A Rauch PJ Chudnovskiy A Berger C. Origins of tumor-associated macrophages and neutrophils. Proc Natl Acad Sci USA. (2012) 109:2491–6. 10.1073/pnas.1113744109 22308361 PMC3289379

[63] Hoeffel G Ginhoux F. Fetal monocytes and the origins of tissue-resident macrophages. Cell Immunol. (2018) 330:5–15. 10.1016/j.cellimm.2018.01.001 29475558

[64] Perdiguero EG Geissmann F. The development and maintenance of resident macrophages. Nat Immunol. (2016) 17:2–8. 10.1038/ni.3341 26681456 PMC4950995

[65] Zhu Y Herndon JM Sojka DK Kim KW Knolhoff BL Zuo C. Tissue-resident macrophages in pancreatic ductal adenocarcinoma originate from embryonic hematopoiesis and promote tumor progression. Immunity. (2017) 47:323–38.e6. 10.1016/j.immuni.2017.07.014 28813661 PMC5578409

[66] Ginhoux F Guilliams M. Tissue-resident macrophage ontogeny and homeostasis. Immunity. (2016) 44:439–49. 10.1016/j.immuni.2016.02.024 26982352

[67] Hoeffel G Ginhoux F. Ontogeny of tissue-resident macrophages. Front Immunol. (2015) 6:486. 10.3389/fimmu.2015.00486 26441990 PMC4585135

[68] Ginhoux F Greter M Leboeuf M Nandi S See P Gokhan S. Fate mapping analysis reveals that adult microglia derive from primitive macrophages. Science. (2010) 330:841–5. 10.1126/science.1194637 20966214 PMC3719181

[69] Munro DAD Hughes J. The origins and functions of tissue-resident macrophages in kidney development. Front Physiol. (2017) 8:837. 10.3389/fphys.2017.00837 29118719 PMC5660965

[70] Bain CC Hawley CA Garner H Scott CL Schridde A Steers NJ. Long-lived self-renewing bone marrow-derived macrophages displace embryo-derived cells to inhabit adult serous cavities. Nat Commun. (2016) 7:1–14. 10.1038/ncomms11852 27292029 PMC4910019

[71] Liu Z Gu Y Chakarov S Bleriot C Kwok I Chen X. Fate mapping via Ms4a3-expression history traces monocyte-derived cells. Cell. (2019) 178:1509–25.e19. 10.1016/j.cell.2019.08.009 31491389

[72] Li D Ji H Niu X Yin L Wang Y Gu Y. Tumor-associated macrophages secrete CC-chemokine ligand 2 and induce tamoxifen resistance by activating PI3K/Akt/mTOR in breast cancer. Cancer Sci. (2020) 111:47–58. 10.1111/cas.14230 31710162 PMC6942430

[73] Chen Z Feng X Herting CJ Garcia VA Nie K Pong WW. Cellular and molecular identity of tumor-associated macrophages in glioblastoma. Cancer Res. (2017) 77:2266–78. 10.1158/0008-5472.CAN-16-2310 28235764 PMC5741820

[74] Zhu Y Herndon JM Sojka DK Kim KW Knolhoff BL Zuo C. Tissue-Resident macrophages in pancreatic ductal adenocarcinoma originate from embryonic hematopoiesis and promote tumor progression. Immunity. (2017) 47:323–38.e6. 10.1016/j.immuni.2017.08.018 28813661 PMC5578409

[75] Loyher PL Hamon P Laviron M Meghraoui-Kheddar A Goncalves E Deng Z. Macrophages of distinct origins contribute to tumor development in the lung. J Exp Med. (2018) 215:2536–53. 10.1084/jem.20180534 30201786 PMC6170177

[76] Zhang I Alizadeh D Liang J Zhang L Gao H Song Y. Characterization of arginase expression in glioma-associated microglia and macrophages. PloS ONE. (2016) 11:e0165118. 10.1371/journal.pone.0165118 27936099 PMC5147798

[77] Frankenberger M Hofer TP Marei A Dayyani F Schewe S Strasser C. Transcript profiling of CD16-positive monocytes reveals a unique molecular fingerprint. Eur J Immunol. (2012) 42:957–74. 10.1002/eji.201141907 22531920

[78] Hamers AAJ Dinh HQ Thomas GD Marcovecchio P Blatchley A Nakao CS. Human monocyte heterogeneity as revealed by high-dimensional mass cytometry. Arterioscler Thromb Vasc Biol. (2019) 39:25–36. 10.1161/ATVBAHA.118.311022 30580568 PMC6697379

[79] Gordon S Taylor PR. Monocyte and macrophage heterogeneity. Nat Rev Immunol. (2005) 5:953–64. 10.1038/nri1733 16322748

[80] Ingersoll MA Spanbroek R Lottaz C Gautier EL Frankenberger M Hoffmann R. Comparison of gene expression profiles between human and mouse monocyte subsets. Blood. (2010) 115:e10–9. 10.1182/blood-2009-07-235028 19965649 PMC2810986

[81] Martinez FO Helming L Milde R Varin A Melgert BN Draijer C. Genetic programs expressed in resting and IL-4 alternatively activated mouse and human macrophages: similarities and differences. Blood. (2013) 121:E57–69. 10.1182/blood-2012-06-436212 23293084

[82] Laviron M Boissonnas A. Ontogeny of tumor-associated macrophages. Front Immunol. (2019) 10:1799. 10.3389/fimmu.2019.01799 31417566 PMC6684758

[83] Wolf AA Yanez A Barman PK Goodridge HS. The ontogeny of monocyte subsets. Front Immunol. (2019) 10:1642. 10.3389/fimmu.2019.01642 31379841 PMC6650567

[84] Sica A Mantovani A. Macrophage plasticity and polarization: *in vivo* veritas. J Clin Invest. (2012) 122:787–95. 10.1172/JCI59643 22378047 PMC3287223

[85] Murray PJ Allen JE Biswas SK Fisher EA Gilroy DW Goerdt S. Macrophage activation and polarization: nomenclature and experimental guidelines. Immunity. (2014) 41:339–40. 10.1016/j.immuni.2014.07.009 25035950 PMC4123412

[86] Yunna C Mengru H Lei W Weidong C. Macrophage M1/M2 polarization. Eur J Pharmacol. (2020) 877:173090. 10.1016/j.ejphar.2020.173090 32234529

[87] Sanchez-Martin L Estecha A Samaniego R Sanchez-Ramon S Vega MA Sanchez-Mateos P. The chemokine CXCL12 regulates monocyte-macrophage differentiation and RUNX3 expression. Blood. (2011) 117:88–97. 10.1182/blood-2009-12-258186 20930067

[88] Candido JB Morton JP Bailey P Campbell AD Karim SA Jamieson T. CSF1R(+) macrophages sustain pancreatic tumor growth through T cell suppression and maintenance of key gene programs that define the squamous subtype. Cell Rep. (2018) 23:1448–60. 10.1016/j.celrep.2018.03.131 29719257 PMC5946718

[89] Saung MT Muth S Ding D Thomas DL Blair AB Tsujikawa T. Targeting myeloid-inflamed tumor with anti-CSF-1R antibody expands CD137+effector T-cells in the murine model of pancreatic cancer. J Immunother Cancer. (2018) 6:118. 10.1186/s40425-018-0435-6 30424804 PMC6234697

[90] Li M Li M Yang Y Liu Y Xie H Yu Q. Remodeling tumor immune microenvironment via targeted blockade of PI3K-γ and CSF-1/CSF-1R pathways in tumor associated macrophages for pancreatic cancer therapy. J Control Release. (2020) 321:23–35. 10.1016/j.jconrel.2020.02.011 32035193

[91] Pyonteck SM Akkari L Schuhmacher AJ Bowman RL Sevenich L Quail DF. CSF-1R inhibition alters macrophage polarization and blocks glioma progression. Nat Med. (2013) 19:1264–72. 10.1038/nm.3337 24056773 PMC3840724

[92] Sluijter M van der Sluis TC van der Velden PA Versluis M West BL van der Burg SH. Inhibition of CSF-1R supports T-cell mediated melanoma therapy. PloS ONE. (2014) 9:e104230. 10.1371/journal.pone.0104230 25110953 PMC4128661

[93] Evans J Giles A Reid C Kaplan R CSF-1R inhibition blocks rhabdomyoscarcoma metastasis by polarizing macrophage differentiation. Cancer Res. (2015) 75:4126—6. 10.1158/1538-7445.AM2015-4126

[94] Beatty GL Chiorean EG Fishman MP Saboury B Teitelbaum UR Sun WJ. CD40 agonists alter tumor stroma and show efficacy against pancreatic carcinoma in mice and humans. Science. (2011) 331:1612–6. 10.1126/science.1198443 21436454 PMC3406187

[95] Poh AR Ernst M. Targeting macrophages in cancer: from bench to bedside. Front Oncol. (2018) 8:49. 10.3389/fonc.2018.00049 29594035 PMC5858529

[96] Kowal J Kornete M Joyce JA. Re-education of macrophages as a therapeutic strategy in cancer. Immunotherapy. (2019) 11:677–89. 10.2217/imt-2018-0156 31088236

[97] Tripathi C Tewari BN Kanchan RK Baghel KS Nautiyal N Shrivastava R. Macrophages are recruited to hypoxic tumor areas and acquire a pro-angiogenic M2-polarized phenotype via hypoxic cancer cell derived cytokines oncostatin M and eotaxin. Oncotarget. (2014) 5:5350–68. 10.18632/oncotarget.2110 25051364 PMC4170629

[98] Chanmee T Ontong P Konno K Itano N. Tumor-associated macrophages as major players in the tumor microenvironment. Cancers. (2014) 6:1670–90. 10.3390/cancers6031670 25125485 PMC4190561

[99] Kumar V Gabrilovich DI. Hypoxia-inducible factors in regulation of immune responses in tumour microenvironment. Immunology. (2014) 143:512–9. 10.1111/imm.12380 25196648 PMC4253499

[100] Zeng W Liu P Pan W Singh SR Wei Y. Hypoxia and hypoxia inducible factors in tumor metabolism. Cancer Lett. (2015) 356 (Pt. A):263–7. 10.1016/j.canlet.2014.01.032 24508030

[101] Osinsky S Bubnovskaya L Ganusevich I Kovelskaya A Gumenyuk L Olijnichenko G. Hypoxia, tumour-associated macrophages, microvessel density, VEGF and matrix metalloproteinases in human gastric cancer: interaction and impact on survival. Clin Transl Oncol. (2011) 13:133–8. 10.1007/s12094-011-0630-0 21324802

[102] Laoui D Van Overmeire E Di Conza G Aldeni C Keirsse J Morias Y. Tumor hypoxia does not drive differentiation of tumor-associated macrophages but rather fine-tunes the M2-like macrophage population. Cancer Res. (2014) 74:24–30. 10.1158/0008-5472.CAN-13-1196 24220244

[103] Henze AT Mazzone M. The impact of hypoxia on tumor-associated macrophages. J Clin Invest. (2016) 126:3672–9. 10.1172/JCI84427 27482883 PMC5096805

[104] Casazza A Laoui D Wenes M Rizzolio S Bassani N Mambretti M. Impeding macrophage entry into hypoxic tumor areas by Sema3A/Nrp1 signaling blockade inhibits angiogenesis and restores antitumor immunity. Cancer Cell. (2013) 24:695–709. 10.1016/j.ccr.2013.11.007 24332039

[105] Ye LY Chen W Bai XL Xu XY Zhang Q Xia XF. Hypoxia-induced epithelial-to-mesenchymal transition in hepatocellular carcinoma induces an immunosuppressive tumor microenvironment to promote metastasis. Cancer Res. (2016) 76:818–30. 10.1158/0008-5472.CAN-15-0977 26837767

[106] Park JY Sung JY Lee J Park YK Kim YW Kim GY. Polarized CD163+tumor-associated macrophages are associated with increased angiogenesis and CXCL12 expression in gastric cancer. Clin Res Hepatol Gas. (2016) 40:357–65. 10.1016/j.clinre.2015.09.005 26508473

[107] Hu JM Liu K Liu JH Jiang XL Wang XL Yang L. The increased number of tumor-associated macrophage is associated with overexpression of VEGF-C, plays an important role in Kazakh ESCC invasion and metastasis. Exp Mol Pathol. (2017) 102:15–21. 10.1016/j.yexmp.2016.12.001 27939650

[108] Jensen TO Schmidt H Moller HJ Hoyer M Maniecki MB Sjoegren P. Macrophage markers in serum and tumor have prognostic impact in american joint committee on cancer stage I/II melanoma. J Clin Oncol. (2009) 27:3330–7. 10.1200/JCO.2008.19.9919 19528371

[109] Ch'ng ES Sharif SET Jaafar H. In human invasive breast ductal carcinoma, tumor stromal macrophages and tumor nest macrophages have distinct relationships with clinicopathological parameters and tumor angiogenesis. Virchows Arch. (2013) 462:257–67. 10.1007/s00428-012-1362-4 23283409

[110] Ying L Yan F Meng Q Yuan X Yu L Williams BRG. Understanding immune phenotypes in human gastric disease tissues by multiplexed immunohistochemistry. J Transl Med. (2017) 15:206. 10.1186/s12967-017-1311-8 29025424 PMC5639762

[111] Thommen DS Koelzer VH Herzig P Roller A Trefny M Dimeloe S. A transcriptionally and functionally distinct PD-1(+) CD8(+) T cell pool with predictive potential in non-small-cell lung cancer treated with PD-1 blockade. Nat Med. (2018) 24:994–1004. 10.1038/s41591-018-0057-z 29892065 PMC6110381

[112] Hofman P Badoual C Henderson F Berland L Hamila M Long-Mira E. Multiplexed Immunohistochemistry for molecular and immune profiling in lung cancer-just about ready for prime-time? Cancers. (2019) 11:283. 10.3390/cancers11030283 30818873 PMC6468415

[113] Blair TA Michelson AD Frelinger AL. Mass cytometry reveals distinct platelet subtypes in healthy subjects and novel alterations in surface glycoproteins in glanzmann thrombasthenia. Sci Rep. (2018) 8:10300. 10.1038/s41598-018-28211-5 29985398 PMC6037710

[114] Rahman AH Lavin Y Kobayashi S Leader A Merad M. High-dimensional single cell mapping of cerium distribution in the lung immune microenvironment of an active smoker. Cytom B Clin Cytom. (2018) 94:941–5. 10.1002/cyto.b.21545 28734132 PMC6289862

[115] Fu W Wang W Li H Jiao Y Weng J Huo R. High dimensional mass cytometry analysis reveals characteristics of the immunosuppressive microenvironment in diffuse astrocytomas. Front Oncol. (2020) 10:78. 10.3389/fonc.2020.00078 32117733 PMC7010913

[116] Brummelman J Haftmann C Núñez NG Alvisi G Mazza EMC Becher B. Development, application and computational analysis of high-dimensional fluorescent antibody panels for single-cell flow cytometry. Nat Protoc. (2019) 14:1946–69. 10.1038/s41596-019-0166-2 31160786

[117] Hartmann FJ Bendall SC. Immune monitoring using mass cytometry and related high-dimensional imaging approaches. Nat Rev Rheumatol. (2020) 16:87–99. 10.1038/s41584-019-0338-z 31892734 PMC7232872

[118] Hung JH Weng Z Analysis of microarray and RNA-seq expression profiling data. Cold Spring Harb Protoc. (2017) 2017:191–6. 10.1101/pdb.top093104 27574194

[119] Haque A Engel J Teichmann SA Lonnberg T. A practical guide to single-cell RNA-sequencing for biomedical research and clinical applications. Genome Med. (2017) 9:75. 10.1186/s13073-017-0467-4 28821273 PMC5561556

[120] Chen G Ning BT Shi TL. Single-cell RNA-Seq technologies and related computational data analysis. Front Genet. (2019) 10:317. 10.3389/fgene.2019.00317 31024627 PMC6460256

[121] Yu PJ Lin W. Single-cell transcriptome study as big data. Genom Proteom Bioinf. (2016) 14:21–30. 10.1016/j.gpb.2016.01.005 26876720 PMC4792842

[122] Stahl PL Salmen F Vickovic S Lundmark A Navarro JF Magnusson J. Visualization and analysis of gene expression in tissue sections by spatial transcriptomics. Science. (2016) 353:78–82. 10.1126/science.aaf2403 27365449

[123] Achim K Vergara HM Pettit JB. Spatial transcriptomics: constructing a single-cell resolution transcriptome-wide expression atlas. In: Gaspar I, editor. RNA Detection: Methods and Protocols. New York, NY: Springer New York (2018). p. 111–25. 10.1007/978-1-4939-7213-5_7 29130193

[124] Wong K Navarro JF Bergenstråhle L Ståhl PL Lundeberg J. ST spot detector: a web-based application for automatic spot and tissue detection for spatial transcriptomics image datasets. Bioinformatics. (2018) 34:1966–8. 10.1093/bioinformatics/bty030 29360929

[125] Fernández Navarro J Lundeberg J Ståhl PL. ST viewer: a tool for analysis and visualization of spatial transcriptomics datasets. Bioinformatics. (2019) 35:1058–60. 10.1093/bioinformatics/bty714 30875427

[126] Gubin MM Esaulova E Ward JP Malkova ON Runci D Wong P. High-dimensional analysis delineates myeloid and lymphoid compartment remodeling during successful immune-checkpoint cancer therapy. Cell. (2018) 175:1014–30.e19. 10.1016/j.cell.2018.09.030 30343900 PMC6501221

[127] Zhang QM He Y Luo N Patel SJ Han YJ Gao RR. Landscape and dynamics of single immune cells in hepatocellular carcinoma. Cell. (2019) 179:829–45.e20. 10.1016/j.cell.2019.10.003 31675496

[128] Moncada R Barkley D Wagner F Chiodin M Devlin JC Baron M. Integrating microarray-based spatial transcriptomics and single-cell RNA-seq reveals tissue architecture in pancreatic ductal adenocarcinomas. Nat Biotechnol. (2020) 38:333–42. 10.1038/s41587-019-0392-8 31932730

[129] Cao JL Yang X Li JP Wu H Li P Yao ZQ. Screening and identifying immune-related cells and genes in the tumor microenvironment of bladder urothelial carcinoma: based on TCGA database and bioinformatics. Front Oncol. (2020) 9:1533. 10.3389/fonc.2019.01533 32010623 PMC6974676

[130] Sun JL Li S Lu X Feng JB Cai TJ Tian M. Identification of the differentially expressed protein biomarkers in rat blood plasma in response to gamma irradiation. Int J Radiat Biol. (2020) 96:748–58. 10.1080/09553002.2020.1739775 32149567

[131] Huang DW Sherman BT Tan Q Kir J Liu D Bryant D. DAVID bioinformatics resources: expanded annotation database and novel algorithms to better extract biology from large gene lists. Nucleic Acids Res. (2007) 35:W169–75. 10.1093/nar/gkm415 17576678 PMC1933169

[132] Ying L Yan F Meng Q Yu L Yuan X Gantier MP. PD-L1 expression is a prognostic factor in subgroups of gastric cancer patients stratified according to their levels of CD8 and FOXP3 immune markers. Oncoimmunology. (2018) 7:e1433520. 10.1080/2162402X.2018.1433520 29872566 PMC5980489

[133] Williams CB Yeh ES Soloff AC. Tumor-associated macrophages: unwitting accomplices in breast cancer malignancy. NPJ Breast Cancer. (2016) 2:15025. 10.1038/npjbcancer.2015.25 26998515 PMC4794275

[134] Anfray C Ummarino A Andon FT Allavena P. Current strategies to target tumor-associated-macrophages to improve anti-tumor immune responses. Cells. (2019) 9:46. 10.3390/cells9010046 31878087 PMC7017001

[135] Chen Y Song Y Du W Gong L Chang H Zou Z. Tumor-associated macrophages: an accomplice in solid tumor progression. J Biomed Sci. (2019) 26:78. 10.1186/s12929-019-0568-z 31629410 PMC6800990

